# Regrowth of Microcosm Biofilms on Titanium Surfaces After Various Antimicrobial Treatments

**DOI:** 10.3389/fmicb.2019.02693

**Published:** 2019-11-25

**Authors:** Qi Han, Yaling Jiang, Bernd W. Brandt, Jingmei Yang, Yu Chen, Mark J. Buijs, Wim Crielaard, Lei Cheng, Dongmei Deng

**Affiliations:** ^1^State Key Laboratory of Oral Disease, National Clinical Research Center for Oral Diseases, Department of Pathology, Sichuan University, Chengdu, China; ^2^Department of Preventive Dentistry, Academic Center for Dentistry Amsterdam (ACTA), University of Amsterdam and VU University Amsterdam, Amsterdam, Netherlands; ^3^State Key Laboratory of Oral Disease, National Clinical Research Center for Oral Diseases, Department of Cariology and Endodonics, West China Hospital of Stomatology, Sichuan University, Chengdu, China; ^4^State Key Laboratory of Oral Disease, National Clinical Research Center for Oral Diseases, Department of Periodontics, West China Hospital of Stomatology, Sichuan University, Chengdu, China

**Keywords:** peri-implantitis, antimicrobials, biofilm regrowth, 16S rDNA, microcosm, oral microbiome

## Abstract

**Objectives:** Our aim of this work was to investigate the regrowth of implant-related biofilms after various antimicrobial treatments *in vitro*.

**Methods:** Saliva-derived microcosm biofilms were grown on titanium discs in an active attachment model. Treatments including hydrogen peroxide (HP), citric acid (CA), chlorhexidine (CHX), and distilled water (control), at different concentrations, were applied to 2-day biofilms for 1 or 5 min. The viability, lactic acid production, and composition of the biofilms were followed for 3 days. The biofilm composition was analyzed by 16S rDNA amplicon sequencing.

**Results:** The short treatments of CA, CHX, and HP resulted in a 2–3 log reduction in biofilm viability and lactic acid production immediately. However, both parameters returned to the pre-treatment level within 2 days due to biofilm regrowth. The alpha diversity of the regrown biofilms in antimicrobial-treated groups tended to decrease, whereas the diversity of those in water-treated group increased. The composition of the regrown biofilms altered compared to those before treatments. *Streptococcus* and *Enterobacteriaceae* were enriched in the regrown biofilms.

**Conclusions:** Although the antimicrobial treatments were efficient, the multi-species biofilms were indeed able to regrow within 2 days. The regrown biofilms display an altered microbial diversity and composition, which in the oral cavity may lead to an aggressive infection.

## Introduction

The use of dental implants has become a popular solution for the successful restoration of missing teeth in recent decades (Moraschini et al., 2015). The annual number of dental implants placed is estimated at 500,000. Despite the high survival rate of dental implants (above 90%) (French et al., 2015), implant failure does occur. One of the main causes is peri-implantitis characterized by inflammation of the peri-implant mucosa and progressive loss of supporting bone (Schwarz et al., 2018). Currently, the main obstacle in the battle against peri-implantitis is the lack of an effective treatment protocol. Since dental biofilm is the principal etiological factor for peri-implantitis, the primary goal of all types of treatments is to eliminate or control the biofilms around the infected implant (Heitz-Mayfield and Mombelli, 2014). Thus, various treatment strategies share one key step: surface decontamination, including mechanical, chemical, and laser decontamination (Romanos et al., 2015). The most-used chemical agents include hydrogen peroxide (HP), citric acid (CA), and chlorhexidine (CHX).

However, results from clinical studies revealed that, although surface decontamination indeed leads to immediate suppression of anaerobic bacteria on the implant surface, this step alone is not sufficient for a successful treatment outcome (de Waal et al., 2013, 2015). Bacterial cells in a biofilm are notorious for their ability to resist antimicrobial treatments, and the special screw shape design of a dental implant further increases the difficulties in biofilm removal (Renvert and Polyzois, 2015). It was suggested that complete elimination of biofilms from a structured material surface is almost impossible (Hoiby et al., 2010; Doring et al., 2012). Consequently, the biofilm cells surviving the anti-microbial treatments can grow to the level of before treatment in a short period of time.

The regrowth of biofilms after antimicrobial treatments has been considered as a critical reason for the persistent biofilm contamination in several fields, including drinking water system contamination (Douterelo et al., 2016) and persistent *Pseudomonas aeruginosa* infection (Haagensen et al., 2017; Müsken et al., 2018). Concerning the regrowth of dental implant-related biofilms, *Staphylococcus epidermidis* (*S. epidermidis*) has been used as the model bacterial species (Gustumhaugen et al., 2014; Wiedmer et al., 2017). The applied antimicrobial agents determined the regrowth of *S. epidermidis* biofilms on rough titanium surfaces, since the regrowth was observed within 24 h only after 3% HP and HP-titanium dioxide treatments, but not after 0.2% CHX treatment (Wiedmer et al., 2017). Data from these limited studies indicate that the regrowth of biofilms after antimicrobial treatment can be an important outcome parameter when evaluating an antimicrobial agent.

Peri-implantitis is caused by a polymicrobial biofilm infection. Using open-ended DNA sequencing techniques, it was found that the peri-implantitis microbiota is a complex microbial community, containing 100–200 bacterial species (Lafaurie et al., 2017). A multi-species biofilm exhibits enhanced antibiotic resistance as compared to a single-species biofilm (Hall and Mah, 2017). Hence, the regrowth of these biofilms after antimicrobial treatment can be more prominent than that of single-species biofilms. Furthermore, it was shown that the bacterial composition and function of regrown gut microbiota could be affected by the types of antibiotics applied (Perez-Cobas et al., 2013).

The aim of this article was to investigate the viability, metabolic activity, and microbial composition of biofilms regrown after three different chemical decontamination treatments in an *in vitro* saliva-derived biofilm model. Sandblasted acid-etched (SLA) titanium surfaces were used as substrata. The three antimicrobial agents tested were CA, CHX, and HP, all of which are commonly used for surface decontamination during peri-implantitis treatments.

## Materials and Methods

### Microcosm Biofilm Formation

An Amsterdam Active Attachment (AAA) Model was assembled with titanium discs (Deng et al., 2009a; Exterkate et al., 2010). The surfaces of all titanium discs (10 mm diameter and 1 mm thickness) were sandblasted and acid etched following the standard protocol (Baoji Titanium Industry, China).

The biofilm growth medium (BMS) was a semi-defined nutrient poor medium (Bao et al., 2015), containing 76 mM K_2_HPO_4_, 15 mM KH_2_PO_4_, 10 mM (NH_4_)_2_SO_4_, 35 mM NaCl, and 2 mM MgSO_4_·7H_2_O, and was supplemented with filter-sterilized vitamins (including 0.04 mM nicotinic acid, 0.1 mM pyridoxine HCl, 0.01 mM pantothenic acid, 1 μM riboflavin, 0.3 μM thiamine HCl, and 0.05 μM D-biotin), amino acids (4 mM L-glutamic acid, 1 mM L-arginine HCl, 1.3 mM L-cysteine HCl, and 0.1 mM L-tryptophan), 0.3% (w/v) yeast extract, and 0.2% sucrose.

To form microcosm biofilms, saliva collected from a single donor was diluted with fresh BMS at a ratio of 1:20 and inoculated into a 24-well culture plate (1.3 ml/well). The plate was then covered with the lid containing SLA discs and incubated anaerobically (10% CO_2_, 10% H_2_, and 80% N_2_) at 37°C. After 8 h, biofilms on titanium discs were washed once in sterile phosphate buffered saline (PBS) to remove the unattached bacterial cells and then transferred to a new 24-well plate containing fresh BMS and incubated for another 16 h. Thereafter, the BMS was refreshed daily after 8 and 16 h. Next, various antimicrobials treatments were applied to 2-day-old microcosm biofilms.

The study was approved by the Medical Ethical Committee of the VU University Medical Center Amsterdam (document number, 2011/236). The donor was informed about the objectives and procedure of the study and has signed an informed consent.

### Antimicrobial Treatments

The 2-day-old microcosm biofilms were transferred to treatment solutions (1.3 ml/well) after being washed once with PBS. The treatment solutions included 40% CA, 0.4% or 0.8% of CHX, 6% or 12% of HP, or distilled water (as a negative control). The durations of the treatments were 1 min for HP, 1 or 5 min for CA, and 5 min for CHX. The CHX or CA treatments were stopped by placing the biofilms in neutralizer solutions for 10 min. The neutralizer solution for CHX contained 30 g lecithin, 30 g polysorbate 80, per liter (pH 7.2), whereas the solution for CA was 300 mM HEPES solution (pH 7.0). After treatments, three biofilm samples (per treatment) were immediately harvested for further analysis (defined as day-3 biofilms). The rest of samples was transferred to fresh BMS and grown further.

### Biofilm Regrowth

**Figure 1:**
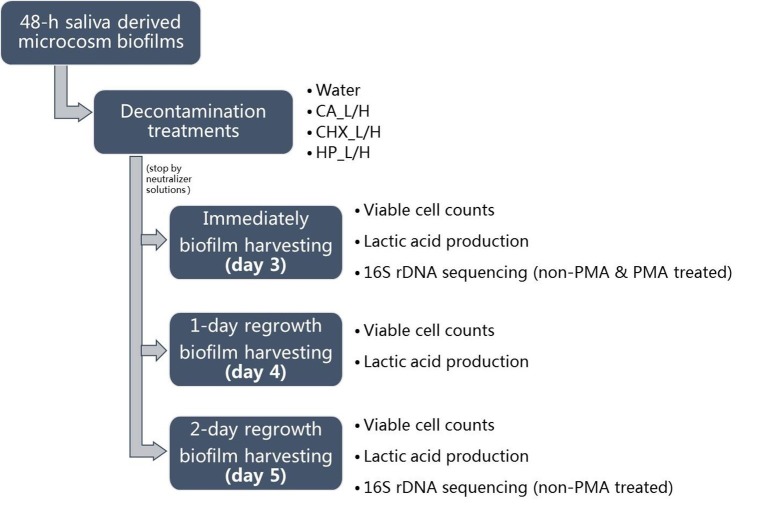
Experimental design of this study. CA, citric acid; CHX, chlorhexidine; HP, hydrogen peroxide; L, low treatment concentration/short duration; H, high treatment concentration/long duration.

For biofilm regrowth, the treated day-3 biofilms were transferred to fresh BMS, which was refreshed every 8 and 16 h as described above. On days 4 and 5, biofilm samples were harvested again for further analysis. The biofilm samples of days 3–5 were analyzed for their lactic acid production and viable cell counts. Those of days 3 and 5 were also subjected to microbial compositional analysis. The schema of biofilm growth, treatments, and regrowth is shown in Figure 1. The experiment was repeated twice. Each experiment contained three biofilm samples per treatment per time point.

### Lactic Acid Production

The biofilms grown on the SLA discs (treated or regrown) were washed with sterile PBS once and transferred to a new 24-well plate containing 1.3 ml/well BMS with 0.4% glucose but without yeast extract and sucrose. The samples were incubated for 1 h at 37°C. The lactic acid concentration in the assay media was measured with an enzymatic spectrophotometric method (Deng et al., 2009b). The principle of this method is based on the enzymatic conversion of L-lactate to pyruvate with the concomitant conversion of NAD to NADH. The increase in absorbance at 340 nm is proportional to the NADH formation.

### Viable Cell Counts

Each titanium disc with biofilms was removed from the clamp and dropped into 2 ml of cysteine peptone water (CPW). The biofilms were dispersed from the discs by vortexing for 30 s, followed by sonication on ice for 2 min at 1 s pulse at an amplitude of 40 W (Vibra Cell; Sonics & Materials Inc., Newtown, CT, USA). The samples (50 μl) were then serially diluted and plated onto trypticase soy agar containing 5% sheep blood, 5 μg/ml hemin, and 1 μg/ml menadione. The plates were incubated anaerobically for 7 days at 37°C before the colony forming units (CFUs) were counted. The remaining sonicated samples (1.95 ml) were further processed either with or without propidium monoazide treatment before microbial compositional analysis.

### Propidium Monoazide Treatment

The Propidium Monoazide (PMA) treatments were only applied to day-3 (treated) biofilm samples. In detail, the sonicated biofilm samples were split into two portions, one portion was incubated with 50 μM PMA (Biotum Inc., Hayward, USA) while the other portion was not. All samples were incubated in dark for 5 min and then exposed to intense light for 2 min using a 650-W halogen lamp at 25 cm from the samples (Nocker et al., 2007). After the treatments, the samples were stored at −80°C for microbiome analysis.

### DNA Extraction, 16S rDNA Sequencing, and Data Processing

DNA extraction, 16S rDNA sequencing, and data processing were performed as described previously (Koopman et al., 2016; Cieplik et al., 2019). Briefly, the genomic DNA (gDNA) of biofilm samples was isolated using phenol bead-beating followed by Agowa nucleic acid isolation (LGC Genomics, Mag mini kit). The V4 region of 16S rDNA was amplified with primers containing respective Illumina adapters and a unique 8-nt index sequence key. After purification, paired-end sequencing of the amplicons was conducted on the Illumina MiSeq platform at the VMC Cancer Center Amsterdam (Amsterdam, the Netherlands) using the Illumina MiSeq reagent kit V3 to generate 251-bp paired-end reads. The cleaned reads were clustered into operational taxonomic units (OTUs) at 97% similarity. The OTU-representative (most abundant) sequence of each OTU was assigned a taxonomy using the ribosomal database project (RDP) classifier and the Human Oral Microbiome Database, sliced to the V4 used region only (HOMD; http://www.homd.org), version 14.51 (Chen et al., 2010). To allow comparison among different samples, the OTU table was subsampled at 8,900 reads per sample before further analysis. The raw sequence data were deposited with the NCBI SRA database as accession PRJNA575687.

### Data Analysis and Statistics

One-way ANOVA was conducted in SPSS version 25 (SPSS Inc., Chicago, IL, USA) to compare the lactic acid production and viable counts of biofilms right after treatment with those after 1- and 2-day regrowth. Differences were considered statistically significant if *p* < 0.05.

PAST (PAlaeontological STatistics) version 3.20 (Hammer et al., 2001) was used to calculate the Shannon diversity and to perform principal component analysis (PCA) and two-way permutational multivariate analysis of variance (PERMANOVA). For the latter two analyses, the OTU table was log_2_-transformed to normalize the data distribution. PERMANOVA was used to determine the influence of regrowth and treatments on microbial composition using the Bray-Curtis similarity index. After removing OTUs with a size less than 100, linear discriminant analysis effect size (LEfSe; Segata et al., 2011) was conducted to uncover OTUs, which were responsible for the shift in composition. The threshold on the logarithmic LDA score was set to 4; otherwise, default settings were used.

## Results

### Viability of Microcosm Biofilms in Time

**Figure 2:**
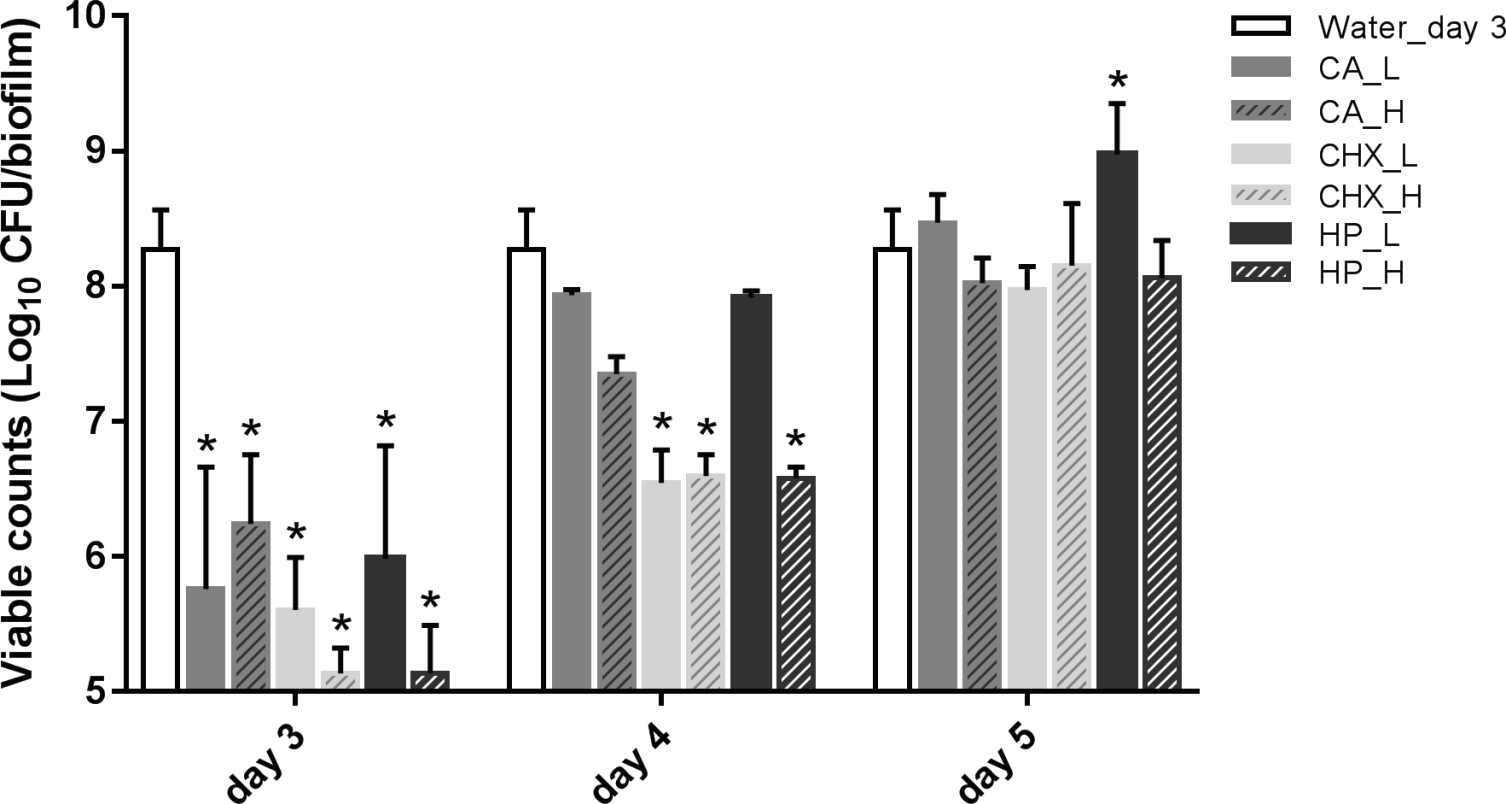
Viable cell counts of biofilms after different decontamination treatments at different time points. Data are presented as mean + SD. * represents statistically significant difference compared with day-3 biofilms of Water control group (Water_day 3) (*p* < 0.05). For clarity, the Water_day 3 data were repeated at days 4 and 5. CA, citric acid; CHX, chlorhexidine; HP, hydrogen peroxide; L, low treatment concentration/short duration; H, high treatment concentration/long duration.

Figure 2 shows the viability of microcosm biofilms in time after various treatments. The treatments were applied on 2-day-old microcosm biofilms, which contained 8.27 ± 0.29 log_10_ CFU/biofilm. All three treatments (CA, CHX, and HP) resulted in significant reductions in biofilm viability, ranging from 2.03 ± 0.51 to 3.14 ± 0.19 log_10_ CFU/biofilm. The viabilities of the treated biofilms started to recover within 1 day, irrespective of the types and concentrations/durations of the treatment. In the low concentrations of CA (CA_L) and HP (HP_L) groups, the biofilm viabilities even completely recovered to the level before treatments. The full recovery of biofilm viability in the rest groups was observed 2 days after the treatments. The biofilm viability in the HP_L group (8.98 ± 0.38 log_10_ CFU/biofilm) was significantly higher than that of before treatment.

In addition, the viable counts of biofilms in the control group increased significantly from 8.27 ± 0.29 log_10_ CFU/biofilm on day 3 (treatment day) to 9.26 ± 0.62 log_10_ CFU/biofilm on day 5 (2-day after treatments). Hence, the biofilm viable count in the HP_L group on day 5 was similar to that of the day-5 control group.

### Changes of Lactic Acid Production of Microcosm Biofilms in Time

**Figure 3:**
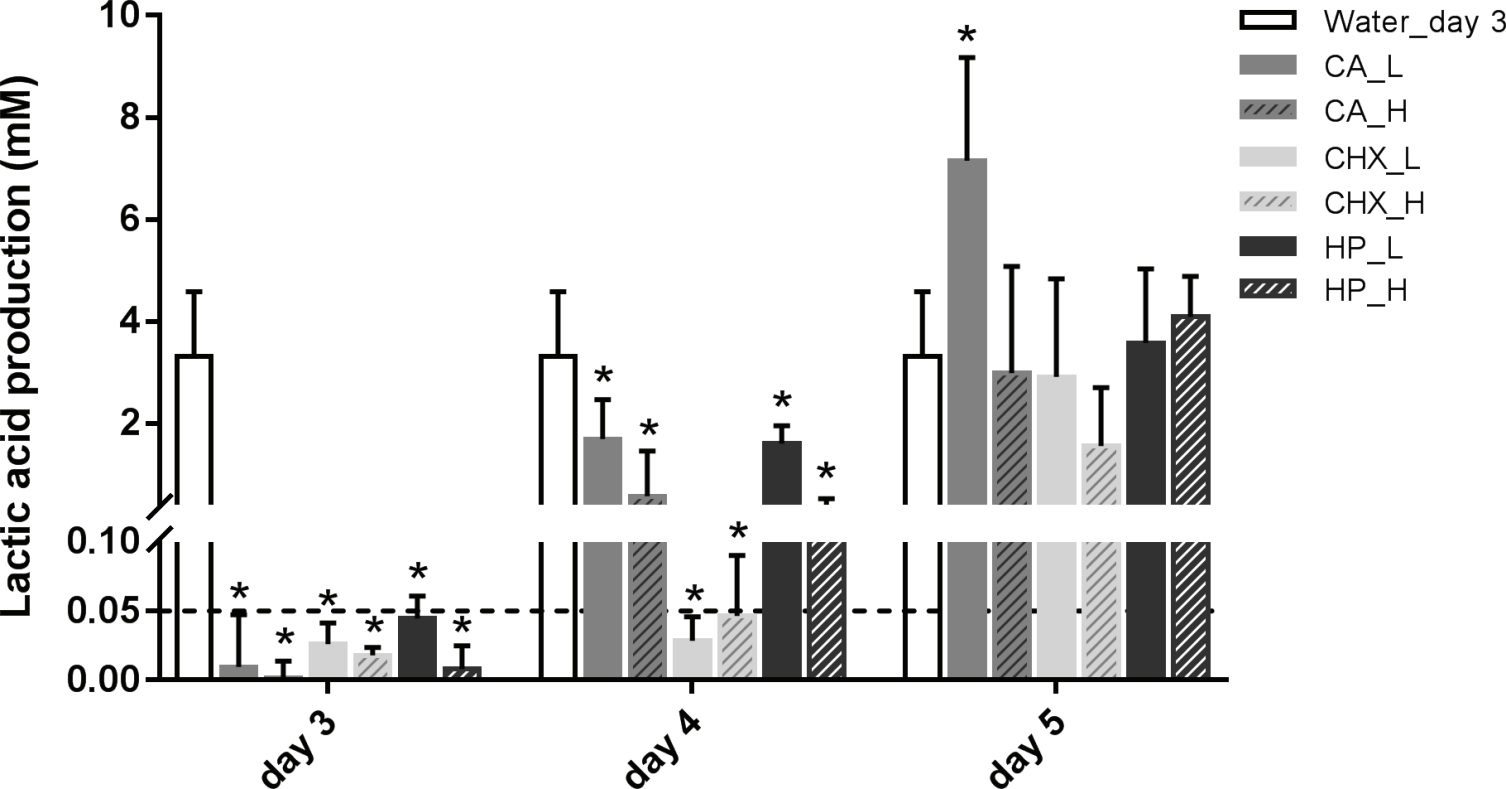
Lactic acid production of biofilms after different decontamination treatments at different time points. Data are presented as mean + SD. * represents statistically significant difference compared with day-3 biofilms of Water group (Water_day 3) (*p* < 0.05). For clarity, the Water_day 3 data were repeated at days 4 and 5. The dashed line indicates the detection limit (0.05 mM). CA, citric acid; CHX, chlorhexidine; HP, hydrogen peroxide; L, low treatment concentration/short duration; H, high treatment concentration/long duration.

The level of lactic acid production by the biofilms was quantified to indicate the metabolic activity of the biofilms. Generally, the changes in lactic acid production of the biofilms showed similar trend as biofilm viability: it dropped right after the treatments and began to recover after 1 day (Figure 3). Only the speed of the recovery seemed to be slower than that of the biofilm viability. One day after treatments, the lactic acid production of all groups was still significantly lower than before treatment. In the control group, the lactic acid production of the biofilms doubled from 3.31 ± 1.27 mM on day 3 to 7.26 ± 1.33 mM on day 5.

### Changes of Microbial Profiles in Time

For microbial composition analysis, only the biofilms of days 3 (right after treatments) and 5 (2 days after treatments) were collected and subjected to sequencing analysis. The sequence data used for analysis originate from the samples, which have not been treated with PMA, unless specified otherwise.

**Figure 4:**
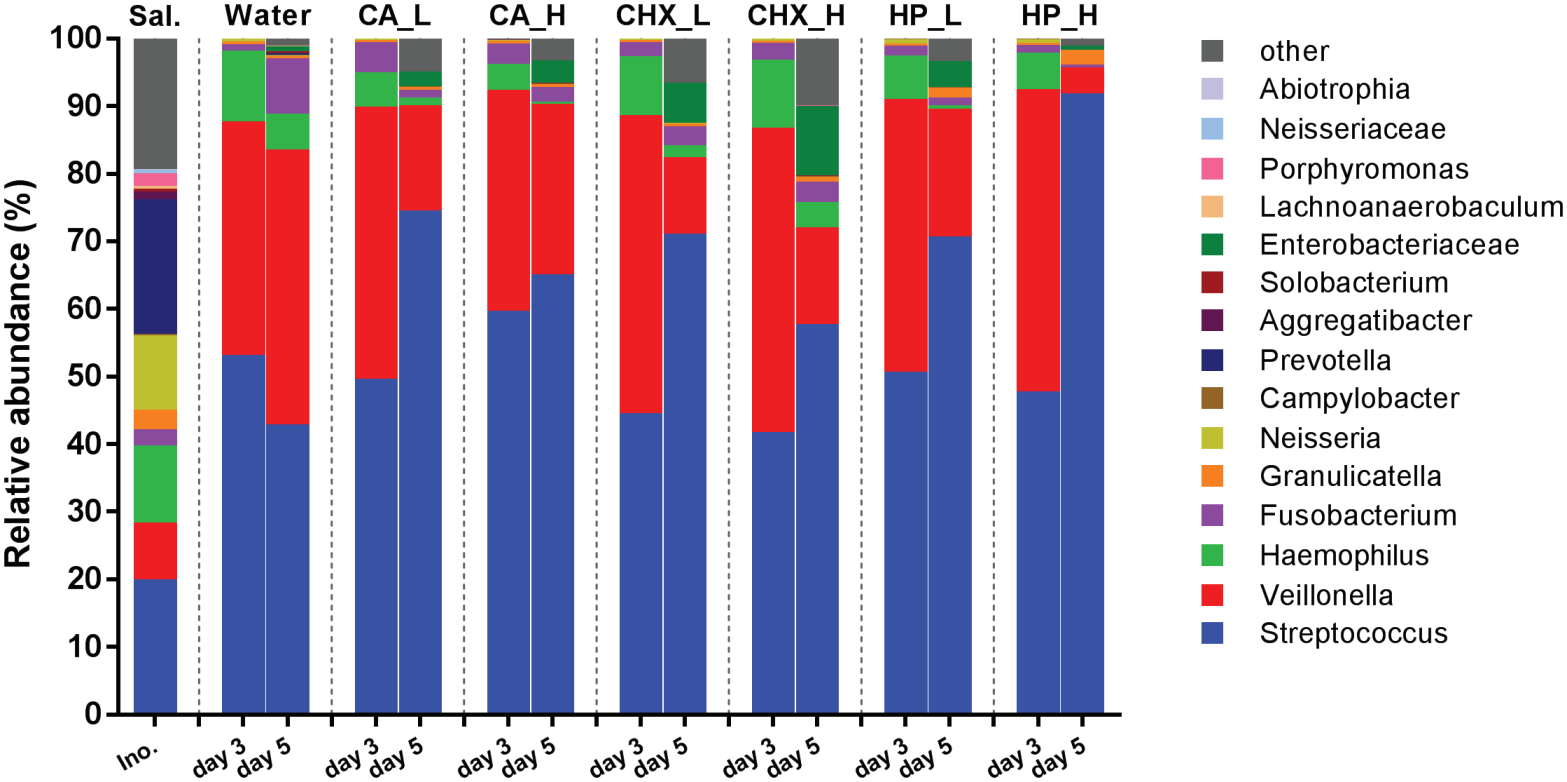
Relative abundance (average of replicates) of top 15 most abundant bacteria genera or higher taxa (remaining genera are grouped as “other”) in saliva inoculum, day-3 and day-5 biofilms. CA, citric acid; CHX, chlorhexidine; HP, hydrogen peroxide; L, low treatment concentration/short duration; H, high treatment concentration /long duration.

Figure 4 shows the average relative abundance of the top 15 bacterial genera or higher taxa in the microcosm biofilms; the saliva inocula are included for comparison. The microbial profiles of the saliva inocula were more diverse than those of the microcosm biofilms (Shannon diversity, *p* = 0.01 for inocula vs. Water_day 3). All microcosm biofilms were dominated by *Streptococcus* and *Veillonella*.

**Figure 5:**
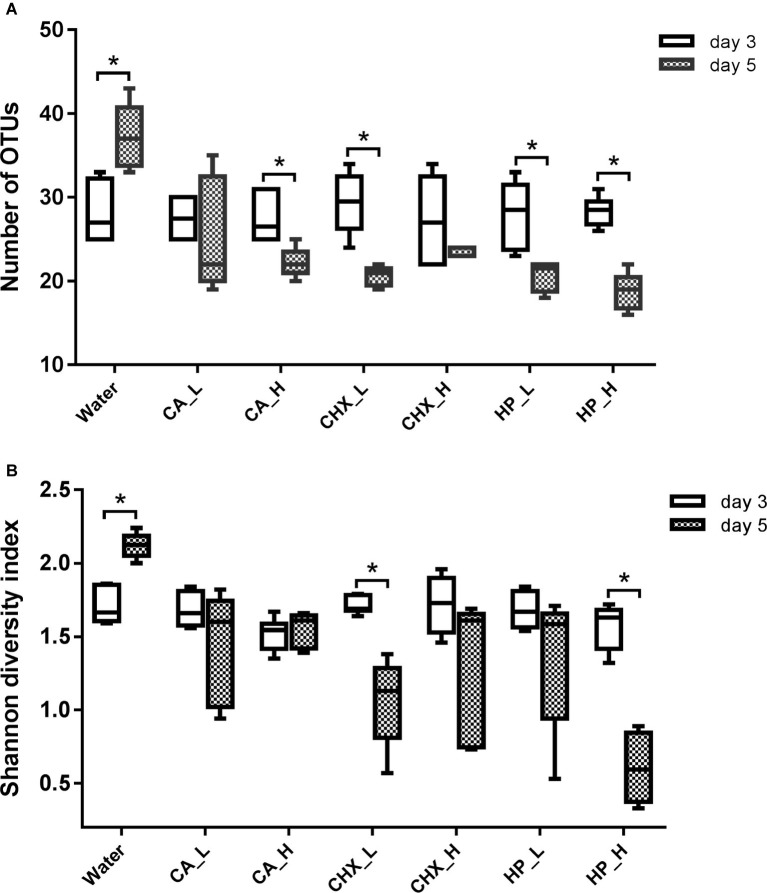
Number of OTUs **(A)** and Shannon diversity index **(B)** of biofilms in different groups. Connector with * indicates statistically significant difference between day-3 and day-5 biofilms within the same treatment group (independent samples *t* test, *p* < 0.05). CA, citric acid; CHX, chlorhexidine; HP, hydrogen peroxide; L, low treatment concentration/short duration; H, high treatment concentration/long duration.

The diversity of the microcosm biofilms, indicated by the number of OTUs and Shannon diversity index, was further analyzed. Figure 5 shows that the number of OTUs of regrown biofilms on day 5 was significantly lower than that on day 3, except for the CA_L and CHX_H groups. The Shannon diversity showed a similar trend, but the reduction was less evident: only the CHX_L and HP_L groups had a significant reduction. Interestingly, the diversity of the biofilms in the control group changed in the opposite direction. Significantly higher diversity of these biofilms on day 5 was observed.

### Changes in Ordination of Microcosm Biofilms in Time

**Figure 6:**
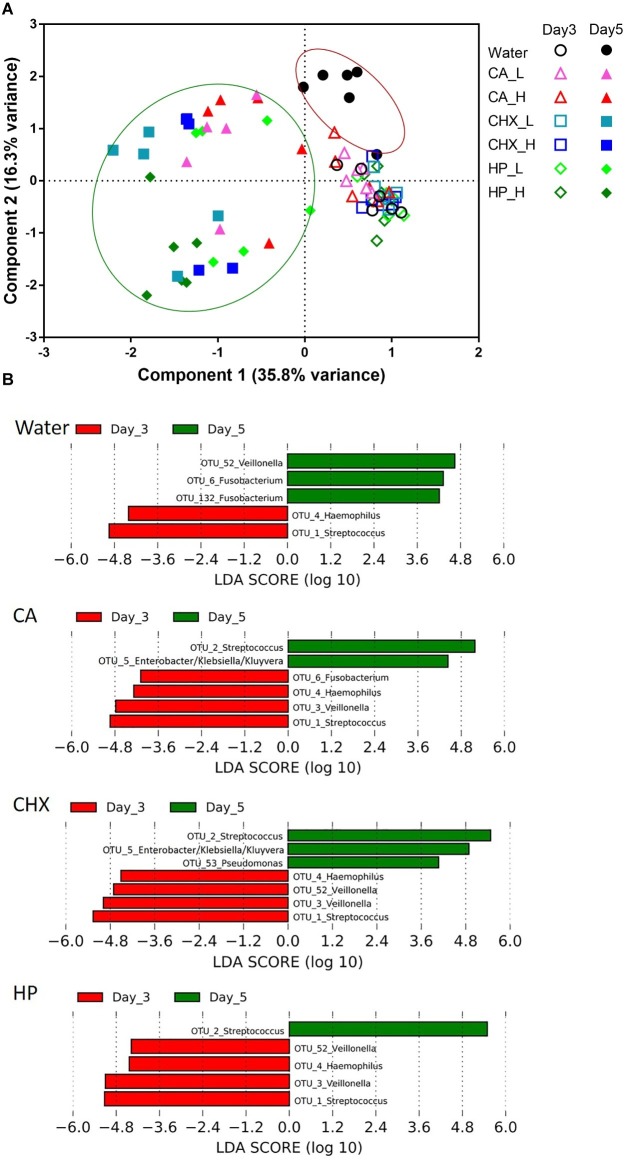
**(A)** Principal component analysis (PCA) plots of day-3 (*open symbols*) and day-5 (*filled symbols*) biofilms in all groups. Biofilms of day 5 were encircled into two parts to visualize the different shift direction between water control group (*red line*) and other groups (*green line*). **(B)** Operational taxonomic units (OTUs) that were differentially abundant between day-3 (*red bars*) and day-5 (*green bars*) biofilms in each group ranked by the effect size in linear discriminant analysis effect size (LEfSe). The species-level taxonomies of the most abundant sequence for the OTUs with the same genus name are: OTU1: *Streptococcus salivarius/vestibularis*; OTU2: *Streptococcus oralis/mitis*; OTU3: *Veillonella parvula*; OTU52: *Veillonella atypica/dispar*; OTU6: *Fusobacterium periodonticum*; OTU132: *Fusobacterium nucleatum* subsp. *polymorphum/*sp*. oral taxon 203*. CA, citric acid; CHX, chlorhexidine; HP, hydrogen peroxide; L, low treatment concentration/short duration; H, high treatment concentration/long duration.

The PCA plot in Figure 6A revealed a clear shift in the microbial composition of the regrown biofilms (day 5) compared to those of the biofilms right after treatments (day 3). The direction of the shifts, however, seemed to be treatment-type dependent. On day 5, the biofilm composition in the control group (*encircled in the red line*) shifted on the *y*-axis (component 2, explaining 16.3% variance) toward the upper direction, whereas those of the actual treatment groups (*encircled in the green line*) shifted on the *x*-axis (component 1, explaining 35.8% variance). Two-way PERMANOVA analysis confirmed that various treatments indeed led to significantly differential treatment-type dependent biofilm composition shifts (*p* = 0.0001, *F* = 3.28).

The data obtained from day-5 biofilms were also analyzed with one-way PERMANOVA. Overall, the composition of the regrown biofilms was significantly affected by the types of treatments (*p* = 0.001, *F* = 6.55). The pairwise comparison (Table 1) shows that the biofilm composition in the control group and the HP_H group was significantly different (or marginally different) from each other and from the other treatment groups.

**Table 1:** Bonferroni corrected *p*-values (in the upper triangle, marked in gray) and *F*-values (in the lower triangle) from pairwise comparison in one-way PERMANOVA statistical analysis. Biofilms of day 5 in various treatment groups were compared.

Group	Water	CA_L	CA_H	CHX_L	CHX_H	HP_L	HP_H
Water		0.044*	0.050	0.055	0.048^*^	0.044^*^	0.046^*^
CA_L	8.789		1.000	0.132	0.334	0.099	0.048^*^
CA_H	7.559	1.526		0.143	0.172	1.000	0.046^*^
CHX_L	19.18	3.355	5.514		1.000	0.183	0.038^*^
CHX_H	12.01	3.647	4.293	1.360		1.000	0.046^*^
HP_L	14.02	2.775	1.960	3.622	2.349		0.147
HP_H	26.97	5.535	6.924	4.913	5.417	4.098	

* *Together with bold font indicates statistically significant difference (p < 0.05)*.

*CA, citric acid; CHX, chlorhexidine; H, high treatment concentration/duration; HP, hydrogen peroxide; L, low treatment concentration/duration*.

LEfSe analysis was used to identify specific bacterial species those abundance changed after biofilm regrowth (Figure 6B). Although each OTU contains a mixture of species, the taxonomy of the OTU’s most abundant sequence is given below for disambiguation. The abundance of OTU3 (*Veillonella parvula*) was significantly reduced in all antimicrobial treatment groups whereas increased in the control group. The abundance of OTU6 (*Fusobacterium periodonticum*) was also significantly increased in the control group, but its reduction was only significant in CA treatment group. Furthermore, all three antimicrobial treatments led to the increase in the abundance of OTU2 (*Streptococcus oralis/mitis*). In CA and CHX groups, the increase of OTU5 (*Enterobacter/Klebsiella/Kluyvera*) was also observed. In all groups, the abundance of OTU4 (*Haemophilus parainfluenzae*) and OTU1 (*Streptococcus salivarius*/*vestibularis*) decreased on day 5 as compared to day 3.

### The Effect of Propidium Monoazide Treatment on Microcosm Biofilms

PMA is a photo-reactive DNA-binding agent that preferentially binds to the DNA of dead cells through defective cell membranes (Nocker et al., 2006). Consequently, the PMA-modified DNA cannot be amplified by PCR. Only the biofilm samples of day 3 were subjected to PMA treatment.

**Figure 7:**
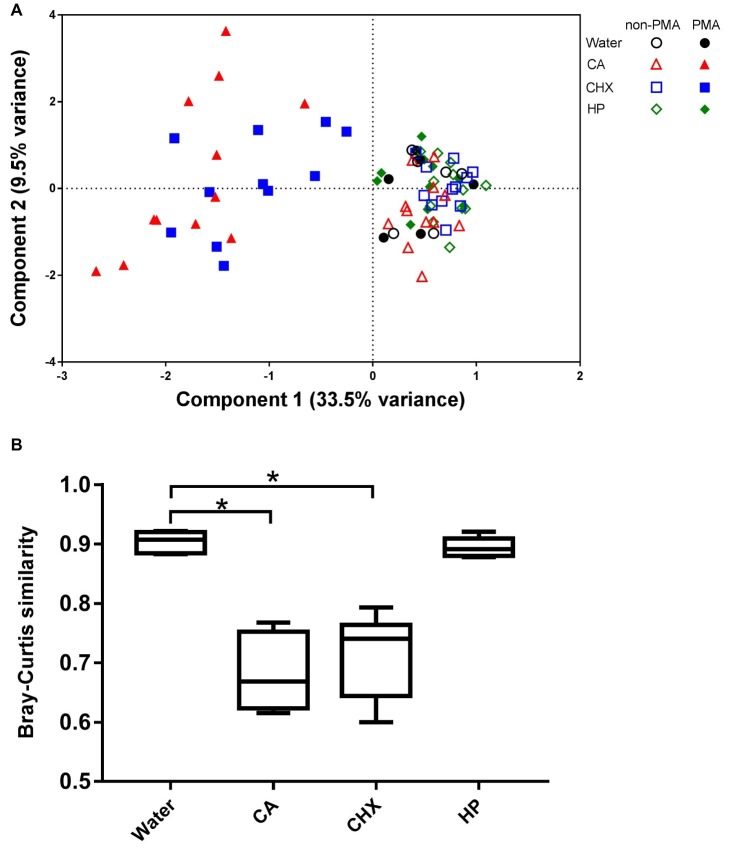
The effect of PMA treatment on the microbial composition of the biofilms. **(A)** PCA plot of non-PMA and PMA treated biofilms. **(B)** Comparison of Bray-Curtis similarity indices between the non-PMA and PMA treated biofilms per decontamination group. * indicates statistically significant difference between the connected two groups (*p* < 0.05). CA, citric acid; CHX, chlorhexidine; HP, hydrogen peroxide.

The PCA plot (Figure 7A) shows that the addition of PMA only led to shifts in the biofilm composition of CA and CHX groups but not in those of HP groups. The first component could explain 33.5% of the variance of the shift of CA and CHX groups on *x*-axis. A comparison of the Bray-Curtis similarity index by independent samples *t* test confirmed that PMA treatment significantly changed the biofilm compositions in the CA and CHX groups but not in the HP group (Figure 7B).

## Discussion

In this study, we investigated biofilm regrowth after different antimicrobial treatments, using a model in which saliva-derived microcosm biofilms were grown on SLA titanium surfaces. Our results showed that the metabolic activity (lactic acid production) and viability of all biofilms were able to recover to the level of before treatment within 2 days, even though both parameters displayed up to 99.9% reduction by various antimicrobial treatments. However, the diversity and the composition of these regrown biofilms differed from those before the treatment. The antimicrobial treatments reduced the diversity and caused a shift in microbial composition of the biofilms.

The rate of biofilm regrowth depends on several factors, such as treatment efficacy, nutrient availability, and species richness of the biofilms. The multi-species biofilms in this study began to regrow within 1 day, much faster than the regrowth speed of a single-species *S. epidermidis* biofilm, which was treated with 0.2% CHX. No regrowth of the *S. epidermidis* biofilm was observed after 24 h (Wiedmer et al., 2017). However, another study (Jing et al., 2019) reported that it took 11 weeks for the biomass of a multi-species biofilm to regrow to the pre-treatment level. The difference in biofilm regrowth between Jing’s study and ours may be explained by the very high concentration of CHX (2%) and the low biofilm medium refreshing frequency (once per week) used in Jing’s study. We observed that a low biofilm medium refreshing frequency reduced the growth speed of biofilms (data not shown). Despite various regrowth speed, results from *in vitro* studies indicated that the residual biofilms remaining after antimicrobial treatments were able to regrow to the pre-treatment level eventually. Current clinical treatments of peri-implantitis do not include a specific strategy targeting biofilm regrowth, except for systemic antibiotics prescription after implant surface decontamination. However, several studies (Carcuac et al., 2016; Hallstrom et al., 2017) have shown that the adjunctive use of systemic antibiotics treatment does not improve the clinical outcome. Likely, the antibiotics do not sufficiently suppress localized biofilm regrowth. A better strategy, which controls biofilm regrowth, is necessary.

Our data show that the application of antimicrobial treatments reduced the species richness of the regrown biofilms and led to shifted microbial profiles. This is in line with the previous findings in gut microbiota after the administration of antibiotics (Antonopoulos et al., 2009; Jakobsson et al., 2010; Zaura et al., 2015). According to the ecological theory, species-rich communities in general are healthier and able to deal with disturbance better than species-poor communities (Levine and D'antonio, 1999; Reese and Dunn, 2018). So far, decreased microbial diversity has been linked with many diseases such as Inflammatory Bowel Diseases, skin diseases, and obesity (Lozupone et al., 2012; Fuentes et al., 2018). It was shown that this decreased diversity may potentially increase susceptibility to aggressive bacterial infections like *Clostridioides difficile* or vancomycin-resistant *Enterococcus* bacteremia (Hunter et al., 2010). In the current study, CA and CHX treatments resulted in the enrichment of *Enterobacteriaceae*, which are known for antibiotics resistance in the regrown biofilms. Further research will characterize the regrown biofilms in antimicrobial resistance.

Initially, PMA was used in this study to exclude DNA of dead cells in the microbiome profiles of the treated samples, since this agent has been successfully applied in microbiome studies (Exterkate et al., 2014). To our surprise, we discovered that the PMA-treatment efficacy depended on the type of antimicrobial agent applied. In theory, PMA treatment should result in a shift in microbial composition as compared to the samples without PMA treatment due to the removal of DNA from dead cells. However, this shift was only observed in CA and CHX-treated samples but not in HP-treated samples, even though the viability and lactic acid production of the biofilms in the HP groups were clearly reduced. Therefore, we chose not to include the data from PMA-treated samples into final microbiota analysis with regard to the day 3 and day 5 comparisons.

Interestingly, the microcosm biofilms in the control group on day 5 showed increased viable cell counts and lactic acid production, increased diversity, and higher abundance of *Veillonella* (OTU52) as compared to those on day 3. This evidence indicates that the biofilms on day 3 had not yet reached a mature steady state. Data from previous studies hint that it might take 14 days for an *in vitro* microcosm biofilm to reach steady state with respect to CFUs and microbial composition (Kistler et al., 2015; Cieplik et al., 2019). In this study, we only examined the regrowth pattern of 2-day-old biofilms. In the follow-up studies, it will be of interest to explore the regrowth pattern of more mature biofilms or the biofilms grown in an animal model, which can better mimic the *in vivo* situation than an *in vitro* model. Furthermore, the microcosm biofilms were grown from the saliva inoculum of one donor. Cieplik et al. (2019) have shown that individual donor determined the composition of the microcosm biofilms. Therefore, it is useful to investigate the effects of different individual donors on biofilm regrowth in the future.

In conclusion, the results from this *in vitro* study provide evidence that a multi-species biofilm is able to regrow within 2 days after effective antimicrobial treatments. The regrown biofilms show reduced diversity and altered composition. In the oral cavity, these altered/new multi-species biofilms may actually lead to a more aggressive and complicated infection. The data from this study also underline the importance of biofilm regrowth control for a successful re-osseointegration.

## Data Availability Statement

The datasets generated for this study can be found in the Sequence Read Archive (SRA) database as accession PRJNA575687.

## Author Contributions

QH and YJ collected and analyzed the data and drafted the paper. BB and MB processed the sequencing samples, analyzed the sequencing data, interpreted the data, and revised the paper critically. WC, LC, and DD designed the study, analyzed the data, and revised the paper critically. All authors approved the final version to be published and agreed to be accountable for all aspects of the work.

### Conflict of Interest

The authors declare that the research was conducted in the absence of any commercial or financial relationships that could be construed as a potential conflict of interest.
